# Interventional Planning for Endovascular Revision of a Lateral Tunnel Fontan: A Patient-Specific Computational Analysis

**DOI:** 10.3389/fphys.2021.718254

**Published:** 2021-08-13

**Authors:** Yunus Ahmed, Christopher Tossas-Betancourt, Pieter A. J. van Bakel, Jonathan M. Primeaux, William J. Weadock, Jimmy C. Lu, Jeffrey D. Zampi, Arash Salavitabar, C. Alberto Figueroa

**Affiliations:** ^1^Department of Cardiac Surgery, University of Michigan, Ann Arbor, MI, United States; ^2^Department of Vascular Surgery, Utrecht University, Utrecht, Netherlands; ^3^Department of Biomedical Engineering, University of Michigan, Ann Arbor, MI, United States; ^4^Department of Radiology, University of Michigan, Ann Arbor, MI, United States; ^5^Department of Pediatrics, University of Michigan, Ann Arbor, MI, United States; ^6^Department of Surgery, University of Michigan, Ann Arbor, MI, United States

**Keywords:** Fontan revision, endovascular repair, interventional planning, computational fluid dynamics, hypoplastic left heart syndrome, congenital heart defect

## Abstract

**Introduction:**

A 2-year-old female with hypoplastic left heart syndrome (HLHS)-variant, a complex congenital heart defect (CHD) characterized by the underdevelopment of the left ventricle, presented with complications following single ventricle palliation. Diagnostic work-up revealed elevated Fontan pathway pressures, as well as significant dilation of the inferior Fontan pathway with inefficient swirling flow and hepatic venous reflux. Due to the frail condition of the patient, the clinical team considered an endovascular revision of the Fontan pathway. In this work, we performed a computational fluid dynamics (CFD) analysis informed by data on anatomy, flow, and pressure to investigate the hemodynamic effect of the endovascular Fontan revision.

**Methods:**

A patient-specific anatomical model of the Fontan pathway was constructed from magnetic resonance imaging (MRI) data using the cardiovascular modeling software CardiovasculaR Integrated Modeling and SimulatiON (CRIMSON). We first created and calibrated a pre-intervention 3D-0D multi-scale model of the patient’s circulation using fluid-structure interaction (FSI) analyses and custom lumped parameter models (LPMs), including the Fontan pathway, the single ventricle, arterial and venous systemic, and pulmonary circulations. Model parameters were iteratively tuned until simulation results matched clinical data on flow and pressure. Following calibration of the pre-intervention model, a custom bifurcated endograft was introduced into the anatomical model to virtually assess post-intervention hemodynamics.

**Results:**

The pre-intervention model successfully reproduced the clinical hemodynamic data on regional flow splits, pressures, and hepatic venous reflux. The proposed endovascular repair model revealed increases of mean and pulse pressure at the inferior vena cava (IVC) of 6 and 29%, respectively. Inflows at the superior vena cava (SVC) and IVC were each reduced by 5%, whereas outflows at the left pulmonary artery (LPA) and right pulmonary artery (RPA) increased by 4%. Hepatic venous reflux increased by 6%.

**Conclusion:**

Our computational analysis indicated that the proposed endovascular revision would lead to unfavorable hemodynamic conditions. For these reasons, the clinical team decided to forgo the proposed endovascular repair and to reassess the management of this patient. This study confirms the relevance of CFD modeling as a beneficial tool in surgical planning for single ventricle CHD patients.

## Introduction

Hypoplastic left heart syndrome (HLHS), is a complex single ventricle congenital heart defect (CHD), characterized by the underdevelopment of the left heart, including left atrium, mitral valve, left ventricle, and aorta (Noonan and Nadas, 1958). HLHS and/or HLHS-variants are estimated to affect 1,025 babies born in the United States each year (Mai et al., 2019). In-hospital mortality rate is between 1 and 2%, while current data suggests 30-year survival of nearly 85% (Ohye et al., 2016). While management of single ventricle lesions has significantly improved over the last decades, HLHS and/or HLHS-variants remain one of the leading causes of death in neonates with CHD (Barron et al., 2009).

**FIGURE 1:**
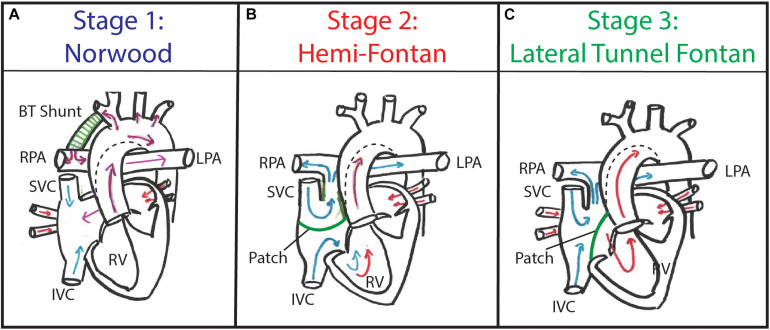
Patients with hypoplastic left heart syndrome (HLHS) typically undergo staged palliation consisting of three consecutive surgeries. **(A)** Stage 1 Norwood procedure consists of an aortic reconstruction and BT shunt implantation. **(B)** Stage 2 Hemi-Fontan procedure consists of superior cavo-pulmonary connection using an intra-cardiac patch. **(C)** Stage 3 Lateral Tunnel Fontan procedure consists of a total cavo-pulmonary connection using a modified intra-atrial patch.

The current treatment paradigm for HLHS and/or HLHS-variants consists of multiple staged reconstructive surgeries, aimed at creating a Fontan circulation in which venous blood is redirected into the lungs and oxygenated blood is pumped into the systemic circulation, supported by a single ventricle [i.e., the morphological right ventricle (RV)] (Feinstein et al., 2012). The stage 1 Norwood procedure is typically performed at birth (Figure 1A). Alternatively, a less invasive hybrid Norwood (i.e., patent ductus arteriosus stenting and pulmonary artery banding) can be performed. A stage 2 superior cavo-pulmonary connection, which, depending on patient anatomy and/or institutional preference can either be a bi-directional Glenn or Hemi-Fontan procedure (Figure 1B), is performed at 4–6 months of age. Finally, stage 3 Fontan completion, creating a total cavo-pulmonary connection, is performed is at 14–48 months of age. At our institution the preferred surgical approach for stage 3 Fontan completion in over 90% of cases consists of an intra-atrial lateral tunnel Fontan procedure (Douglas et al., 1999; Hirsch et al., 2008) (Figure 1C).

Up to two-thirds of HLHS and/or HLHS-variants patients require surgical or transcatheter reintervention within 20 years of the initial staged surgeries, underlining the need for adequate lifetime surveillance (van Dorn et al., 2015; Downing et al., 2017; Daley et al., 2020). Notable complications following the multi-stage surgical approach are arrhythmias, enlarged right atrium (RA), prolonged pleural drainage, seizure, and protein-losing enteropathy (PLE) (Petko et al., 2003). While surgical revision of the Fontan pathway can be performed with low morbidity and mortality, minimally invasive therapeutic options in HLHS and/or HLHS-variants patients can be used to treat patients in frail condition (Park et al., 2016). Patient surveillance typically involves regular pediatric cardiology evaluations with echocardiograms and electrocardiograms. In addition, cardiac catheterization is utilized to assess if a patient is a candidate for each subsequent stage of the single ventricle palliation and to evaluate if there are significant anatomic or hemodynamic abnormalities post-surgery.

Computational fluid dynamics (CFD) is a well-established technique that has been widely used to study hemodynamics of cardiovascular diseases (Taylor and Figueroa, 2009), and can be used to assess the hemodynamic effect of planned surgical interventions (Bove et al., 2003; Sundareswaran et al., 2009; de Zélicourt et al., 2011; Restrepo et al., 2015; van Bakel T. M. J. et al., 2018; Trusty et al., 2019; Toma et al., 2020; Tossas-Betancourt et al., 2020; Li et al., 2021; Primeaux et al., 2021). In this work, we evaluated the feasibility of an endovascular repair considered by the pediatric cardiology team at the University of Michigan C.S. Mott Children’s Hospital to treat a 2-year-old patient with single ventricle CHD presenting with a dilated Fontan pathway and associated PLE. CFD tools were combined with clinical data on anatomy, flow, and pressure to construct a pre-intervention fluid-structure interaction (FSI) model. Then, this model was modified by introducing a custom bifurcated endograft to connect the inferior vena cava (IVC) with the superior vena cava (SVC) and pulmonary arteries. Different indices of hemodynamic performance pre- and post-intervention were obtained to assist the clinical team with determining the course of treatment for this patient.

## Materials and Methods

This study was approved by the University of Michigan Institutional Review Board (HUM00155491).

### Patient’s History

A 2-year-old female with complex HLHS-variant single ventricle CHD (double outlet RV, hypoplastic left ventricle and aortic arch, and malposed great arteries) presented at our institution with new-onset failure to thrive, pleural effusions, fluid overload, and hepatomegaly with concern for PLE. Previously, this patient had undergone pulmonary artery band placement in the neonatal period, followed by a Hemi-Fontan procedure at 6 months of age. At 20 months of age, the patient underwent a Lateral Tunnel Fontan procedure. Concomitantly, a fenestration to the RA was created to alleviate pressure in the Fontan pathway, as is common practice at our institution.

As part of the initial work-up for new-onset PLE, an invasive cardiac catheterization was performed, which revealed elevated mean pressures of ∼15 mmHg in the Fontan pathway. The patient was found to have low arterial oxygen saturation (81%) and borderline low cardiac index of 2.4 L/min/m^2^. Angiography showed hepatic venous reflux, and severe dilation of the inferior segment of the Fontan pathway between the IVC and branch pulmonary arteries with inefficient swirling flow.

### Rationale for Proposed Endovascular Repair

Following thorough evaluation of the patient, the dilation in the Fontan pathway was believed to be the cause of the increased pressures, hepatic venous reflux, subsequent hepatomegaly and new-onset PLE. Revision of the Fontan pathway was indicated, aiming to decrease its size, relieve the hepatic venous reflux, and improve flow to the pulmonary arteries. Due to the frail condition of the patient, the clinical team considered open surgical Fontan revision not to be a suitable option. Instead, a minimally-invasive endovascular revision was considered. This approach entails the deployment of a custom-made endograft to redirect blood flow through the Fontan pathway. A collaboration between biomedical engineering and pediatric cardiology aimed to understand the viability of the proposed endovascular approach, by combining CFD tools with the available clinical data.

### Clinical Data Acquisition

A magnetic resonance imaging (MRI) study was performed using a 1.5 T Ingenia scanner (Philips, Best, Netherlands) with the patient under deep sedation. A free-breathing, ECG-gated, respiratory navigator gated, 3D mDIXON (1.4 mm isotropic voxel size) sequence was performed to acquire the vascular anatomy. The diastolic phase of the 3D MRI data was used to reconstruct the cardiovascular anatomy. Cardiac-gated phase-contrast MRI (PC-MRI) was performed at the SVC, IVC, left pulmonary artery (LPA), and right pulmonary artery (RPA). Flow and luminal area waveforms (consisting of 40 phases, voxel size 1.2 mm, slice thickness 6 mm) were processed from the PC-MRI data using the CVI_42_ software (Circle Cardiovascular Imaging, Calgary, AB, Canada). Invasive catheterization was performed to acquire pressure waveforms at the IVC, Fontan pathway, SVC, LPA, and RPA.

### Computational Analysis

**FIGURE 2:**
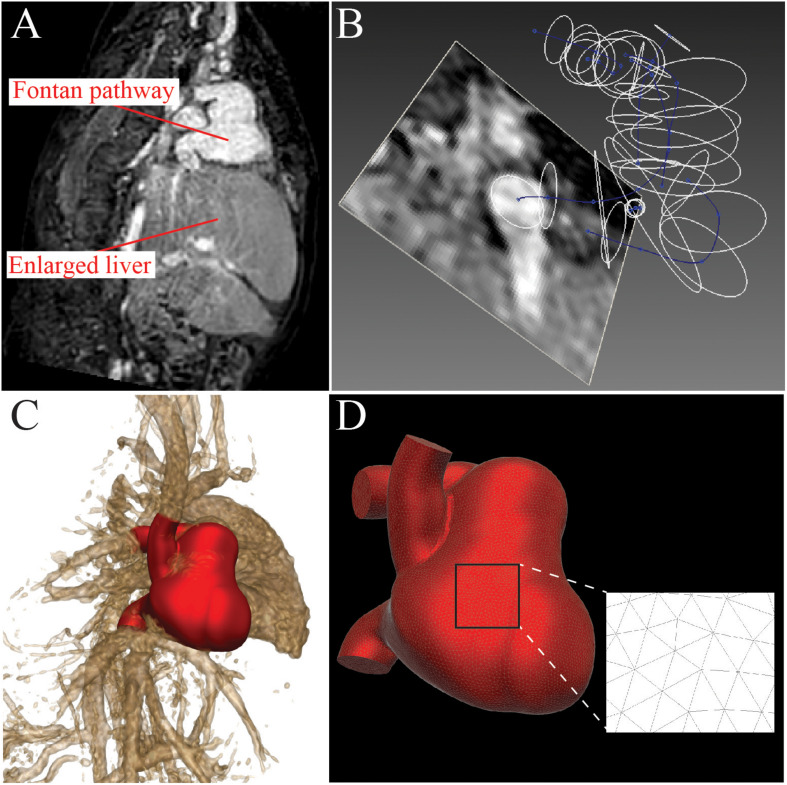
A patient-specific model of the Fontan pathway was constructed from 3D mDIXON MRI data using the CRIMSON software. **(A)** 3D mDIXON MRI data shows a dilated Fontan pathway and enlarged liver. **(B)** 3D path lines were created and 2D contours were made to delineate the vessel walls. **(C)** Vessel contours were combined with a lofting and blending process to create the 3D anatomical model. **(D)** The 3D anatomical model was discretized to create a finite-element mesh consisting of tetrahedral elements.

A pre-intervention 3D model of the Fontan pathway, including the IVC, SVC, LPA, RPA, and fenestration to the RA was constructed (Figure 2A) using the open-source cardiovascular simulation software CRIMSON (Arthurs et al., 2021). Lumen center lines, and 2D segmentations of the vessel lumen (Figure 2B) were used to create a computer aided design (CAD) model of the cardiovascular anatomy using lofting and blending operations (Figure 2C). The CAD model was then discretized into a finite element mesh composed of tetrahedral elements (Figure 2D).

**FIGURE 3:**
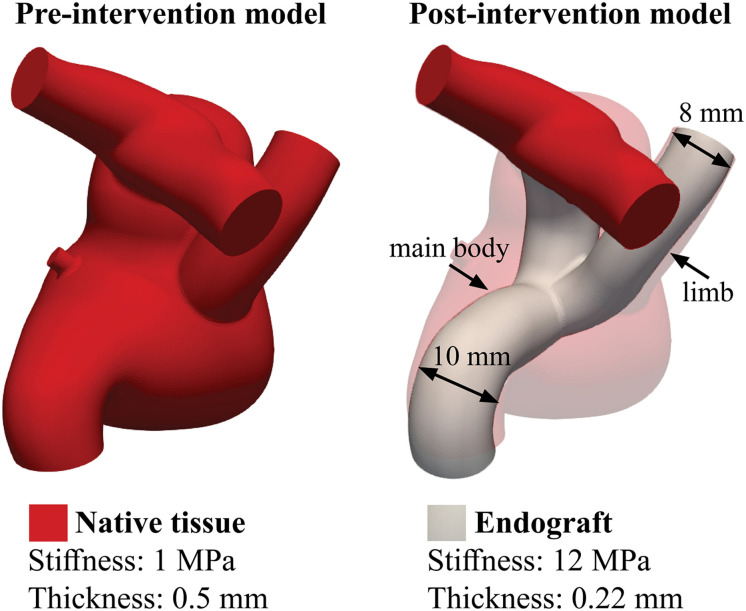
Distribution of stiffness and thickness of the pre- and post-intervention models. The introduction the endograft blocks the fenestration, which was therefore excluded from the post-intervention model.

To model the endovascular repair, the pre-intervention model was modified by introducing a custom bifurcated endograft that connects the IVC, SVC, and pulmonary arteries. The custom bifurcated endograft consist of a 10 mm graft (main body) spanning between the IVC and the ostium of the pulmonary arteries, and an 8 mm limb was deployed into the SVC (Figure 3). The proposed endovascular repair also included excluding the fenestration between the Fontan pathway and the RA. The bifurcated endograft was assigned a stiffness of 12 MPa and thickness of 0.22 mm (Lee and Wilson, 1986).

### Multi-Scale Modeling Approach

**FIGURE 4:**
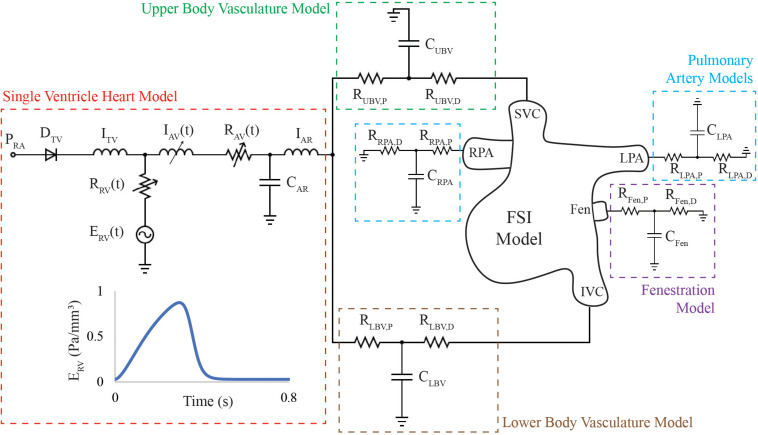
3D-0D open loop model of the HLHS patient includes an FSI model of the Fontan circulation containing venae cavae, pulmonary arteries and fenestration, and lumped-parameter network of models representing the single ventricle heart and vasculature proximal and distal to the FSI model. A single ventricle heart model was placed in series with 3-element Windkessel models representing the upper and lower body vasculature, respectively. Flow is directed into the SVC and IVC and leaves the 3D domain through the LPA, RPA, and fenestration. The parameters of the single ventricle heart model, Windkessel, and wall properties of the FSI model were simultaneously tuned to match patient-specific mean flow, backflow, and pressure data. The patient-specific RV elastance function described the active contraction of the single ventricle heart model.

A 3D-0D open-loop modeling approach was adopted to describe hemodynamics pre- and post-intervention (Figure 4). Lumped-parameter models (LPMs) (0D) were used to represent inflow and outflow boundary conditions (Vignon-clementel et al., 2006). This approach made it possible to simulate pre- and post-intervention conditions without directly imposing any of the measured pressure and flow waveforms. The parameters of the 3D-0D open-loop model are calibrated to reproduce pre-intervention hemodynamic data such as regional flow splits, IVC pressure, and backflow volume per beat, which was believed to contribute to the patient’s hepatomegaly and PLE. These parameters remain unchanged for the post-intervention model. Therefore, any differences in global hemodynamics between the models are the direct consequence of the different geometry and material properties of such model.

For the inflow, a single ventricle heart model (red box) was placed in series with 3-element Windkessel models representing the upper body vasculature (UBV, green box), and lower body vasculature (LBV, brown box), respectively. For the outflows, 3-element Windkessel models were coupled to the LPA, RPA, representing the pulmonary circulation (blue boxes) and the fenestration (purple box).

The single ventricle heart model contains circuits representing the RA, tricuspid valve (TV), RV, aortic valve (AV), and aortic root (AR). The TV was modeled using a diode and inductor in series. The active contraction of the ventricle was modeled using a “two-Hill” time-varying elastance function (Mynard et al., 2012):

$$E_{R\,V}\,\left(t\right)=k\,\left(\frac{g_{1}}{1+g_{1}}\right)\,\left(\frac{1}{1+g_{2}}\right)+E_{m\,i\,n}$$

where

$$g_{1}={\left(\frac{t}{\tau_{1}}\right)}^{m_{1}},g_{2}={\left(\frac{t}{\tau_{2}}\right)}^{m_{2}},k=\frac{E_{m\,a\,x}-E_{m\,i\,n}}{\max\left[\left(\frac{g_{1}}{1+g_{1}}\right)\,\left(\frac{1}{1+g_{2}}\right)\right]}$$

*k* is a scaling factor used to ensure that the minimum and maximum elastance values of the “two-Hill” elastance match the values of the clinically-measured minimum and maximum elastance. Minimum elastance was calculated by dividing ventricular end-diastolic pressure (7 mmHg) over end-diastolic volume (31 mL), whereas the maximum elastance was calculated by dividing ventricular end-systolic pressure (105 mmHg) over end-systolic volume (16 mL). *m_1* and τ_1_ control the slope and time translation of the ascending portion of the elastance waveform, respectively. *m_2* and τ_2_ control the slope and time translation of the descending portion of the elastance waveform, respectively. The AV was represented through a dynamically-controlled resistor and inductor, see Mynard et al. (2012). The final values of the heart model parameters are reported in Table 1.

**TABLE 1:** Parameter values and units for components of the single ventricle heart model.

**Elastance waveform parameters**							
***E*_*MAX*_ (Pa mm^–3^)**	***E*_*MIN*_ (Pa mm^–3^)**	***T* (s)**	**τ_1_ (s)**	***m*_1_ ()**	**τ_2_ (s)**	***m*_2_ (s)**	

0.875	0.03	0.8	0.4944T	2.6	0.5493T	18	

**Heart model parameters**							
***P*_*RA*_ (Pa)**	***R*_*TV*_ (Pa s mm^–3^)**	***I*_*TV*_ (Pa s^2^ mm^–3^)**	***C*_*AR*_ (Pa mm^–3^)**	***I*_*AR*_ (Pa s^2^ mm^–3^)**			

629	1.1 × 10^–5^	1.0 × 10^–5^	0.5	2.0 × 10^–6^			

**Parameters for I_*AV*_(t), R_*RV*_(t), and R_*AV*_(t)**							
***A*_*ANN*_ (mm^2^)**	***P*_*OPEN*_ (Pa)**	***P*_*CLOSE*_ (Pa)**	***K*_*VO*_ (Pa^–1^ s^–1^)**	***K*_*VC*_ (Pa^–1^ s^–1^)**	***M*_*RG*_ ()**	***M*_*ST*_ ()**	***L*_*EFF*_ (mm)**

60	0	1333	0.2	0.2	0.5	1.0	15

**ρ (g mm^–3^)**	***K*_*S*_ (s mm^–3^)**						

0.00106	1.5 × 10^–6^						

*The time constants are expressed in terms of the cardiac cycle length (*T* = 0.8 s).*

The resistance and capacitance values of these models were iteratively tuned until the mean flow at the IVC, SVC, LPA, RPA, retrograde volume per beat at the IVC, LPA, and RPA, and the pressure waveform at IVC were matched to the available clinical data. The values of the LPM parameters are reported in Table 2.

**TABLE 2:** Calibrated parameter values of Windkessel models.

**Vessel**	***R*_*P*_ (Pa s mm^–3^)**	***C* (mm^3^ Pa^–1^)**	***R*_*D*_ (Pa s mm^–3^)**
Upper vasculature	0.1047	4.0000	0.1684
Lower vasculature	0.1847	0.9000	0.0884
Left pulmonary artery	0.0040	7.1974	0.3995
Right pulmonary artery	0.0054	9.3408	0.2668
Fenestration	0.2517	1.3703	1.4265

Wall deformation of the 3D anatomy was modeled using the coupled momentum method (Figueroa et al., 2006). The stiffness of the 3D model was simultaneously tuned, together with the Windkessel model parameters of the circuits, to match the backflow measured at the IVC, LPA, and RPA within 7% of the clinical data. The calibrated wall stiffness and thickness were 1 MPa and 0.5 mm, respectively (Figure 3).

Pulsatile FSI simulations were conducted using CRIMSON’s Navier-Stokes flow solver on 60 cores of the University of Michigan’s high-performance computing (HPC) cluster ConFlux. Blood was modeled as an incompressible Newtonian fluid with a density of 1.06 g/cm^3^ and a dynamic viscosity of 4.0 Pa s. Simulations were run until cycle-to-cycle periodicity in hemodynamic results was achieved. Mesh refinement was performed using an adaptive field-based technique (Sahni et al., 2006) until the hemodynamic results were independent of mesh size. The reported results of the pre-intervention and post-intervention models use refined meshes consisting of 1,754,964 and 1,813,505 elements, respectively.

### Energy Dissipation

Energy dissipated (*E*_*diss*_) was computed using the energy fluxes (Marsden et al., 2007),

$$E_{d\,i\,s\,s}=E_{i\,n}-E_{o\,u\,t}$$

where

$$E_{i\,n}=\sum_{i}^{N_{i\,n\,l\,e\,t\,s}}Q_{i}\,\left(P_{i}+\frac{1}{2}\,\rho \,V_{i}^{2}\right),$$

$$E_{o\,u\,t}=\sum_{j}^{N_{o\,u\,t\,l\,e\,t\,s}}Q_{j}\,\left(P_{j}+\frac{1}{2}\,\rho \,V_{j}^{2}\right).$$

*N*_*inlets*_ and *N*_*outlets*_ are the number of inlet (SVC and IVC) and outlet (LPA, RPA, and fenestration) faces in the model, respectively. Flow rates (*Q*) were integrated over each face, and velocities (*V*) and pressures (*P*) were spatially averaged over each vessel face. The blood density (ρ) was 1.06 g/cm^3^. The energy efficiency was defined as,

$$E_{e\,f\,f}=\frac{E_{o\,u\,t}}{E_{i\,n}}.$$

Energy dissipated was non-dimensionalized using the formula given by Dasi et al. (2009),

$$e_{d\,i\,s\,s}=\frac{E_{d\,i\,s\,s}}{{}_{0}}$$

where

$$\epsilon_{0}=\frac{\rho \times Q^{3}}{{\text{BSA}}^{2}}.$$

The body surface area (BSA) of the patient was 0.51 m^2^.

## Results

### Pre-intervention Model

**FIGURE 5:**
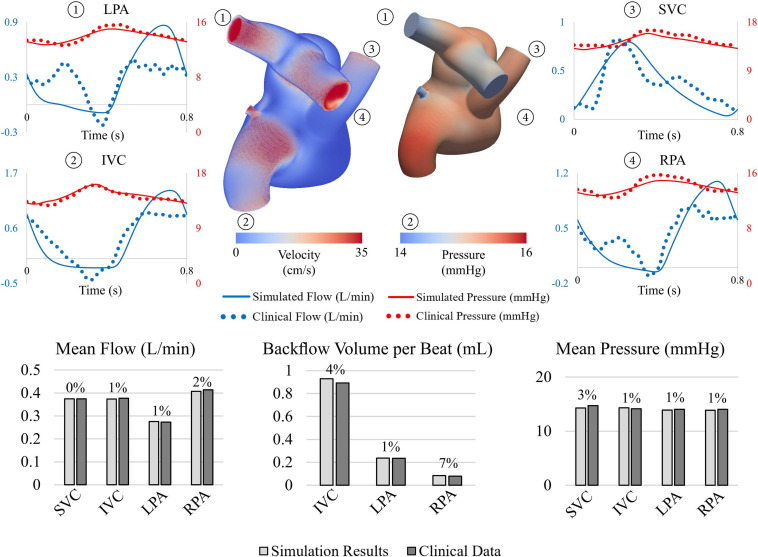
**(Top)** Velocity volume rendering and pressure contour at end systole. Comparison between flow and pressure waveforms at the LPA, IVC, SVC, and RPA show good agreement. **(Bottom)** Comparison between simulated results (light gray) and clinical data (dark gray).

The pre-intervention FSI model successfully reproduced patient-specific hemodynamic data, see Figure 5. Simulation results matched IVC, SVC, LPA, and RPA flow data within 2%. Retrograde volume per beat, a key hemodynamic parameter in understanding the observed symptoms in this patient, was matched within 7% of clinical data acquired at the IVC, LPA, and RPA. Systolic, diastolic, and mean pressures were matched within 5% of clinically acquired pressures at the SVC, IVC, LPA, and RPA.

**FIGURE 6:**
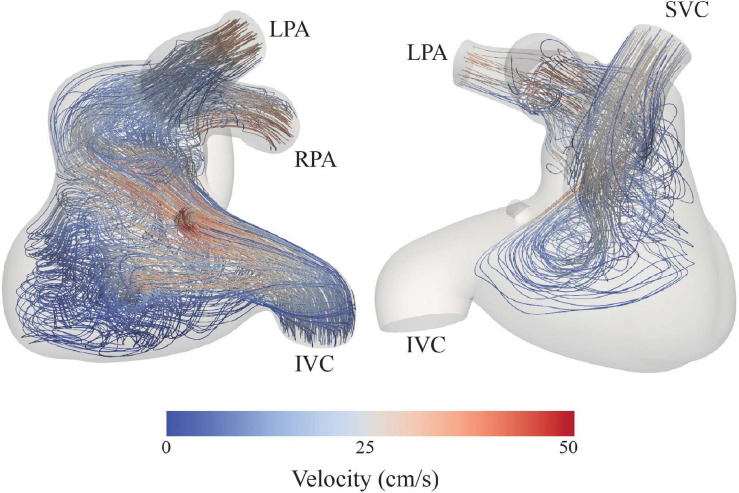
Velocity streamlines depict swirling flow and low velocities in the dilated portion of the Fontan pathway.

The sum of the inlet and outlet energy fluxes was 2.39 × 10^7^ and 2.11 × 10^7^ g mm^2^ s^–3^, respectively. Therefore, the energy flux dissipated in the system was 0.27 × 10^7^ g mm^2^ s^–3^, resulting in an energy efficiency of 88%. The non-dimensionalized energy dissipation was 3.46 × 10^8^. Furthermore, assessment of velocity field streamlines showed re-circulation within the Fontan pathway (Figure 6), specifically in the enlarged pouch, which contribute to the observed dissipation of energy.

### Post-intervention Model

**FIGURE 7:**
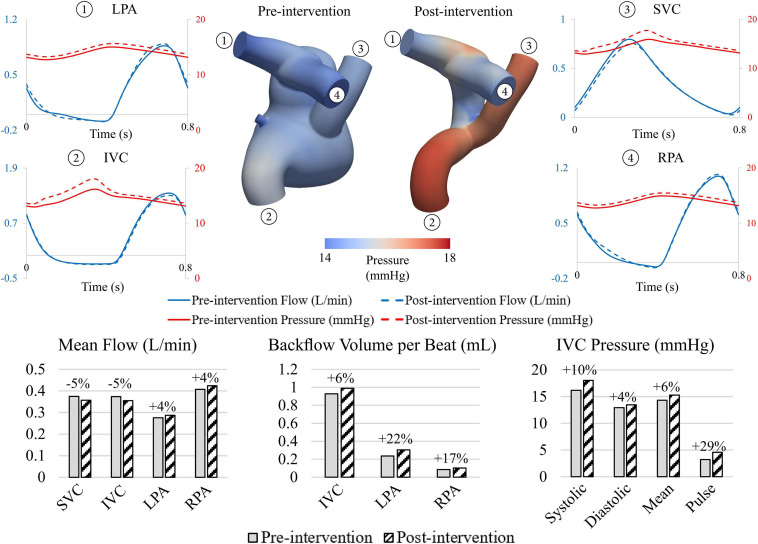
Comparison between pre- and post-intervention hemodynamics. Endovascular intervention led decreases in mean flow and increases in mean pressure at IVC and SVC and increases in backflow at the IVC.

When comparing pre- and post-intervention hemodynamics, the endovascular repair led to a 6 and 7% mean pressure increase and 29 and 43% pulse pressure increase at the IVC and SVC, respectively (Figure 7). Pressures at the pulmonary arteries were increased slightly in the post-intervention model (4% mean pressure increases for both RPA and LPA).

The endograft repair also resulted in a 5% decrease in mean flows at the IVC and SVC. This decrease in mean inflow, together with the increase in pressure, reveals that the proposed endovascular Fontan revision has a larger intrinsic resistance to flow than the pre-intervention anatomy. This higher resistance is likely due to the smaller luminal diameters of the considered endografts. Furthermore, the exclusion of the fenestration in the post-intervention model led to a 4% in mean flow at the pulmonary arteries. The backflow volume per beat at the IVC, which was suspected to contribute to the hepatomegaly, increased by 6%, while a more notable increase in backflow was observed at the pulmonary arteries (17 and 22% increase at the RPA and LPA, respectively).

Energy flux dissipated within the post-intervention model was 54% smaller than in the pre-intervention model (0.13 × 10^7^ vs. 0.27 × 10^7^ g mm^2^ s^–3^). Furthermore, the dimensionless energy flux dissipated within the post-intervention model (1.85 × 10^8^) was 47% smaller than in the pre-intervention model (3.46 × 10^8^). This suggests that the post-intervention model is more efficient in preserving energy from the venae cavae to the pulmonary arteries.

### Clinical Decision Making

Our hemodynamic assessment using CFD revealed that the proposed endovascular Fontan revision failed to reduce the pressure in the Fontan pathway and alleviate hepatic venous reflux. In fact, an increase in backflow volume and pressure was observed in the IVC and pulmonary arteries. Since these outcomes were deemed unfavorable for the patient, the clinical team decided to forgo the proposed intervention.

Besides the predicted limited hemodynamic benefit of a transcatheter Fontan revision in this patient, this procedure would likely be highly technically challenging. Our initial concept was to modify commercially available covered stents and implant these stents in rapid succession to exclude the dilated portion of the Fontan pathway while maintaining flow from the SVC and IVC to the pulmonary arteries. We also considered using bifurcating endovascular grafts. However, these would also likely require customization/modification to fit the anatomy. Given the patient’s age and size, it is unlikely that the peripheral vascular anatomy would accept the typical sheath sizes necessary for endovascular grafts. Lastly, besides limitations in equipment, we need to account for the expected somatic growth of this patient. As the patient grows, we would anticipate redilation of stents would be required and with stent dilation and migration, an endovascular leak could occur. Therefore, technical challenges further steered clinical decision making away from transcatheter Fontan revision in this patient.

## Discussion

Fontan patients are faced with numerous complications during their lifetime, often requiring multiple reinterventions. A comprehensive preoperative assessment, which includes in-depth hemodynamic analysis, is therefore paramount in deciding adequate course of treatment. Patient-specific CFD modeling tools can be used to non-invasively describe the effects of planned interventions. Our group has demonstrated the clinical applicability of advanced computational modeling tools in prospectively aiding surgical planning (van Bakel T. M. J. et al., 2018), as well as evaluating the effect of several surgical interventions to treat cardiovascular disease (Nauta et al., 2017; van Bakel T. M. et al., 2018; Tossas-Betancourt et al., 2020; Primeaux et al., 2021). CFD in surgical planning for single ventricle CHD has been widely used (Troianowski et al., 2011; Haggerty et al., 2013; de Zélicourt and Kurtcuoglu, 2016), where authors successfully validated their post-operative models, built during surgical planning, with clinical data. While these studies employed advanced modeling techniques, such as coupling of 3-element Windkessel models, and imposing pulsatile flow waveforms, the focus of these studies was on energy dissipation and pulmonary flow distribution, which justifies the choice of boundary conditions. However, in our study, we use a heart model at the inlet boundary (Mynard et al., 2012). A heart model allows the virtual intervention to not only affect inlet pressures, but also affect the inlet flow waveforms. The pulsatile nature of our model has been previously shown to result in a more accurate assessment of energy dissipation and hepatic flow distribution (Wei et al., 2018). While work by de Zélicourt and Kurtcuoglu (2016) emphasized that, for prospective surgical planning applications, parameter fitting and validation could be an issue, all of our modeling assumptions have been informed by the available pre-operative clinical data to ensure a proper analysis.

In this work, we explored the feasibility of an endovascular Fontan revision of a 2-year-old CHD patient presenting with complications following Fontan palliation. The patient-specific workflow used in this study combined CFD modeling tools with clinical data to successfully represent the patient hemodynamics and subsequently simulate the proposed endovascular revision. To accurately capture the hemodynamic effects of the proposed endovascular repair, a 3D-0D open-loop modeling approach was adopted. This approach made it possible to simulate pre- and post-intervention conditions without directly imposing any of the measured pressure and flow waveforms. Parameters of the 3D-0D open-loop model were calibrated to reproduce pre-intervention hemodynamic data and remained unchanged for the post-intervention model. Therefore, any differences in global hemodynamics between the models are the direct consequence of the different geometry and material properties of the endograft. While a more complex design of the LPMs could have been employed to represent the different vascular beds, this would have led to a larger number of parameters to be estimated.

The proposed endovascular revision, while resulting in a locally more efficient pathway from the venae cavae to the pulmonary arteries (54% reduction in energy flux dissipated compared to pre-intervention conditions), led to an overall increase in resistance on the venous circulation, which resulted in increases in mean and pulse pressure at the IVC and SVC, and an increase in hepatic venous reflux. These findings are most likely explained by the combination of a decrease in luminal area and increase in structural stiffness following endograft introduction, leading to a loss of compliance in the Fontan pathway. It has previously been demonstrated that introducing a stiff endograft can adversely impact cardiac and arterial hemodynamics (van Bakel et al., 2019). Even though the present work is focused on the Fontan pathway, a low-pressure system, our computational results show increases in pressure, similar to those encountered following TEVAR deployment in the thoracic aorta. These findings underline the importance of adequately assessing the hemodynamic impact of surgical interventions.

Computational tools have promising potential in the field of interventional planning, however, there are several aspects limiting widespread clinical use. First, virtual surgical planning is time consuming and computationally expensive. The analysis performed in this work was completed over a span of several months and required considerable computing power on a dedicated HPC. Second, the operator-dependent nature of the 3D model construction process could potentially result in variability in the geometric model shape and volume. This could subsequently lead to variability in certain hemodynamic indices such as energy dissipation, pressure, and flow. Third, while we were able to virtually assess the performance of the proposed endovascular Fontan repair, we could not validate our findings with post-intervention clinical data. This was due to the fact that the clinical team decided against pursuing the proposed endovascular revision, driven by the results of our analysis and by feasibility issues. Fourth, since the discussed work is an analysis conducted on a single patient, it is difficult to translate these findings to other patients. Finally, this study does not account for hemodynamic adaptions in the system (baroreflex effects, metabolic adaptations) or long-term growth and remodeling, although such CFD applications are currently being developed. Future directions should focus on the developing of semi-automated, user-friendly workflows aimed at providing reliable hemodynamic assessments in the clinical setting. Toward that end, it is important to gather anatomical, flow and pressure assessment in routine patient work-up.

## Data Availability Statement

The raw data supporting the conclusions of this article will be made available by the authors, without undue reservation.

## Ethics Statement

This study was approved by the University of Michigan Institutional Review Board (HUM00155491). Written informed consent from the participants’ legal guardian/next of kin was not required to participate in this study in accordance with the national legislation and the institutional requirements.

## Author Contributions

YA, CT-B, AS, and CF: conceptualization. YA, JP, WW, JL, JZ, and AS: data collection. YA, CT-B, PB, JP, and CF: formal analysis. AS and CF: funding acquisition. WW, JL, JZ, AS, and CF: supervision. YA, CT-B, and CF: writing – original draft. YA, CT-B, PB, JP, WW, JL, JZ, AS, and CF: writing – review and editing. YA, CT-B, PB, JP, WW, JL, JZ, AS, and CF: final approval of article. CF: overall responsibility. All authors contributed to the article and approved the submitted version.

## Conflict of Interest

The authors declare that the research was conducted in the absence of any commercial or financial relationships that could be construed as a potential conflict of interest.

## Publisher’s Note

All claims expressed in this article are solely those of the authors and do not necessarily represent those of their affiliated organizations, or those of the publisher, the editors and the reviewers. Any product that may be evaluated in this article, or claim that may be made by its manufacturer, is not guaranteed or endorsed by the publisher.

## Abbreviations

0D: 0-dimensional
2D: 2-dimensional
3D: 3-dimensional
AR: aortic root
CAD: computer-aided design
CFD: computational fluid dynamics
CHD: congenital heart defect
CRIMSON: CardiovasculaR Integrated Modeling and SimulatiON
FSI: fluid-structure interaction
HLHS: hypoplastic left heart syndrome
HPC: high-performance computing
IVC: inferior vena cava
LBV: lower body vasculature
LPA: left pulmonary artery
LPM: lumped parameter model
MRI: magnetic resonance imaging
PC-MRI: phase-contrast magnetic resonance imaging
PLE: protein-losing enteropathy
RA: right atrium
RPA: right pulmonary artery
RV: right ventricle
SVC: superior vena cava
TEVAR: thoracic endovascular aortic repair
TV: tricuspid valve
UBV: upper body vasculature.
